# The Rheological Studies on Poly(vinyl) Alcohol-Based Hydrogel Magnetorheological Plastomer

**DOI:** 10.3390/polym12102332

**Published:** 2020-10-13

**Authors:** Norhiwani Mohd Hapipi, Saiful Amri Mazlan, U. Ubaidillah, Koji Homma, Siti Aishah Abdul Aziz, Nur Azmah Nordin, Irfan Bahiuddin, Nurhazimah Nazmi

**Affiliations:** 1 Engineering Materials and Structures (eMast) iKohza, Malaysian-Japan International Institute of Technology, Universiti Teknologi Malaysia, Jalan Sultan Yahya Petra, Kuala Lumpur 54100, Malaysia; hiwani87@gmail.com (N.M.H.); sitiaishah.aa@utm.my (S.A.A.A.); nurazmah.nordin@utm.my (N.A.N.); nurhazimah@utm.my (N.N.); 2 Mechanical Engineering Department, Faculty of Engineering, Universitas Sebelas Maret, Jl. Ir. Sutami 36A Kentingan Jebres, Surakarta 57126, Indonesia; 3 International Centre, Tokyo City University, 1 Chrome-28-1 Tamazutmi, Setagaya, Tokyo 158-0087, Japan; khomma@tcu.ac.jp; 4 Mechanical Engineering Department, Vocational College, Universitas Gadjah Mada, Yogyakarta 55281, Indonesia; irfan.bahiuddin@ugm.ac.id

**Keywords:** hydrogel, magnetorheological plastomer, polyvinyl alcohol, rheology, viscoelasticity

## Abstract

The freezing–thawing method has been commonly used in the preparation of polyvinyl alcohol hydrogel magnetorheological plastomer (PVA HMRP). However, this method is complex and time consuming as it requires high energy consumption and precise temperature control. In this study, PVA HMRP was prepared using a chemically crosslinked method, where borax is used as crosslinking agent capable of changing the rheological properties of the material. Three samples of PVA HMRP with various contents of carbonyl iron particles (CIPs) (50, 60, and 70 wt.%) were used to investigate their rheological properties in both steady shear and dynamic oscillation modes. Results showed the occurrence of shear thickening behaviour at low shear rate (γ > 1 s^−1^), where the viscosity increased with the increased of shear rate. Moreover, the storage modulus of the samples also increased increasing the oscillation frequency from 0.1 to 100 Hz. Interestingly, the samples with 50, 60 70 wt.% of CIPs produced large relative magnetorheological (MR) effects at 4916%, 6165%, and 10,794%, respectively. Therefore, the inclusion of borax to the PVA HMRP can offer solutions for a wide range of applications, especially in artificial muscle, soft actuators, and biomedical sensors.

## 1. Introduction

Magnetorheological (MR) materials consist of micron-sized magnetic particles embedded in a carrier matrix, where its rheological properties can be reversibly controlled by the application of the external magnetic field. The materials can be categorized into several groups depending on the type of carrying matrix such as MR fluids, MR elastomers, MR grease, and MR gels [1,2,3,4]. Recently, a new kind of MR material known as MR plastomer (MRP) has gained a great attention from researchers as it is believed to possess high stability and better MR performance [5,6,7]. MRP is prepared by dispersing magnetic particles into a low crosslinking polymer network as a carrying matrix. The carrying matrix of MRP can be grouped into hydrogels, swollen polymer gels and pure polymer gels [8,9]. Amongst them, the preparation of hydrogel MRP (HMRP) was the most facile, economic, and least time-consuming. Hydrophilic polymers (e.g., polyvinyl alcohol (PVA), carrageenan, agar, guar gum, etc.) with softness and flexible characteristics preferred due to the ability of the polymer network to absorb significant volumes of water. HMRP may be rendered as the best candidate for soft actuators such as artificial muscle, vibration absorber and in the application of biomedical sensors due to its elastic and osmotic properties [10,11,12,13]. Moreover, the HMRP could have both solid-like and liquid-like behaviours, which would result in reversible changes in the dynamic modulus [14,15]. Interestingly, carbonyl iron particles (CIPs) trapped within the HMRP matrix are moveable, unlike the MR solid where the CIPs are locked within the matrix phase. Initially, CIPs are randomly suspended within the HMRP forming an isotropic microstructure and are unable to move on their own due to polymer matrix constraints. A mechanism for restructuring of CIPs in HMRP can be explained in relation to the existence of external mechanical force or stimulus-like magnetic field. When the external magnetic field is applied to the material, the CIPs will begin to move by overcoming the matrix constraints and rearrange themselves into an anisotropic microstructure, following the lines of magnetic flux in the HMRP. The anisotropic structure of CIPs will also remain unchanged and can be sustained in the HMRP for several periods when the magnetic field is turned off since the low crosslinking polymer network of the hydrogel matrix preserves the kinetic stability of the CIPs and reduced settling time for the CIPs. However, if any forces are exerted into the HMRP, the CIPs will restructure into isotropic orientation, which proved that the rheological properties of HMRP are reversible.

To date, HMRP materials previously known as magnetic hydrogel have been thoroughly studied in both physical and mechanical aspects, such as compression, tensile, rheological properties, etc. [16,17,18,19]. Amongst all the listed hydrophilic polymer stated before, PVA has been proven to have better mechanical performance. For instance, Mitsumata et al. [16] investigated the magnetic response of magnetic hydrogel based on k-carrageenan. The study showed that the magnetic hydrogel storage modulus changed from ~104 up to 4 MPa when subjected to an external magnetic field of 500 mT. Meanwhile, Negami et al. [17] investigated the mechanical performance for both carrageenan and PVA magnetic hydrogel. The result revealed that PVA gel had a higher yield point of up to 0.8 compared to carrageenan-based which had a lower yield point of 0.35. It proved that PVA has better mechanical toughness than carrageenan. Furthermore, PVA itself has several advantages that are biocompatible and biodegradable, making them very useful for the development of a system for biomedical engineering applications such as artificial cartilage, and biosensors [18,19]. In general, PVA HMRP can be prepared using two methods: physical and chemical crosslinking. The most common approach for preparing hydrogel solution is the use of a physical method known as a freeze-thawing process [20,21,22]. Wu et al. [22], for example, used this method to study the dynamic properties of physically crosslinked PVA HMRP. The maximum MR effect obtained by physically crosslinked PVA HMRP with 70 wt.% of CIPs was up to 230% and the tensile strength was high as 1 MPa. They also studied the stress-shear rate relationship, where the result showed shear-thinning behaviour after fitting with the Herschel–Bulkley model. However, the manufacturing process of physically crosslinked PVA hydrogel has been reported to be complexed and time-consuming. This was because, this method required a high energy consumption and the needed for the accurate control of heating and cooling rates during preparation [23]. By contrast, the chemically crosslinked hydrogel preparation method was much easier and economical with borax being used as one of the chemical crosslinking agents [23,24,25]. Recently, Hapipi et al. [24] studied the solvent effect on the rheological properties of chemically HMRP. They focused on finding the best solvent to reduce desiccation and corrosion of HMRP so that it could last in a long-term operation.

Nonetheless, to the best of our knowledge, systematic understanding of the rheological properties particularly chemically crosslinked PVA HMRP is rarely reported. There is still a lot of uncertainty about the rheological and viscoelastic properties of chemical HMRP. It is imperative to provide useful guidance for the development of new, easy-to-use smart soft materials so that it can be adapted into potential applications. Therefore, more experimental studies should be performed to provide more information on this material as it is still in the development stage. Furthermore, the flow behaviour index (shear thinning/thickening) is one of the important criteria for the researchers to explore more, particularly in the related phenomenon and mechanisms. For example, according to Zhang et al. [26] materials that exhibit shear thickening will demonstrate potential application in energy absorption in ballistic and impact test to be used in anti-impact application. Hence, this paper aims to systematically investigate the rheological and viscoelastic performance of chemically crosslinked PVA HMRP integrated with different CIPs contents, i.e., 50 wt.%, 60 wt.%, and 70 wt.%. Two different test modes, namely a steady shear test and dynamic shear test of chemically crosslinked PVA HMRP under rotational and oscillatory shear rheometry have been studied, respectively. The flow behaviour of PVA HMRP is calculated by steady-state shear testing by fitting the data to the Herschel–Bulkley model. Next, the dynamic viscoelastic measurements of the samples, such as frequency-dependent, absolute MR effect, and relative MR effect, are investigated and discussed based on the results of the oscillatory shear mode tests. Additionally, through a frequency sweep test, the strain-thickening behaviour in dynamic mode of PVA HMRP can be studied.

## 2. Materials and Methods

### 2.1. Materials

PVA with a degree of hydrolysis ≥98% and an average molecular weight of 60,000 g/mol (Merck Company) was used to prepare the precursor solution. Sodium tetraborate decahydrate (borax), 20 Mule Team BoraxTM (drug store) was used as a crosslinking agent. Dimethyl Sulfoxide (DMSO) brand ChemAR was supplied by Systerm Chemicals and deionized water (DI) was used as a solvent. All these three chemicals were used to prepare matrix based PVA HMRP. On the other hand, CIPs with a size of ~5 µm were used as magnetic particles, procured from BASF company (CC type).

### 2.2. Preparation of Chemically Crosslinked Polyvinyl Alcohol Hydrogel Magnetorheological Plastomer (PVA HMRP)

First, PVA HMRP samples were prepared by preparing a 7.5% (*w/v*) PVA solution by diluting 8.1 g of PVA beads into binary mixtures of DI: DMSO (20:80) at 80 °C for 2 h. The magnetic bar was used for a continuous gentle stirring process to ensure the homogeneity of the PVA solution. Next, the solution was cooled to room temperature before CIPs with varying concentrations of 50, 60, and 70 wt.% were added into the solution, individually. The mixture was thoroughly mixed using mechanical stirring for 10 min before the crosslinking agent (borax solution) was added. The borax solution (3% *w/v*) was prepared by dissolving the borax powder in DI water. After the addition of the crosslinking agent, the gelation of PVA HMRP was formed. The as-prepared PVA HMRP was preserved overnight before testing to ensure the uniformity of the PVA HMRP samples were formed. PVA HMRP samples that contained various concentrations 50, 60, and 70 wt.% of CIPs were labelled as HMRP-50, HMRP-60, HMRP-70, respectively.

### 2.3. Structural Characterization and Rheological Studies

A Microsense 7404 vibrating system magnetometer (VSM) was employed to study the magnetic hysteresis loops of PVA HMRP samples with various concentrations of CIPs. The rheological properties of the PVA HMRP samples were determined using a commercial rheometer (Model: MCR 302 Anton Paar, Austria) equipped with an external magneto-controllable accessory, MRD 70/1T. The sample was placed in a parallel plate of 20 mm in diameter with a gap of 1 mm in thickness. Two types of measurement, steady-state rotary shear mode and oscillatory shear mode, were conducted in order to investigate the static and dynamic rheological properties of the samples, respectively. For steady-state rotary shear, the shear rate ranges from 0.001 to 100 s^−1^ at 25 °C. Meanwhile, for oscillatory mode, the shear frequency was carried out from 0.1 to 100 Hz and the strain was kept at 0.03%. During the test, the current varied from 0, 1, 2, and 3 A, which is equivalent to 0, 180, 360, and 540 mT, respectively. Moreover, the MR effect of each sample was calculated from the magnetic flux density sweep test.

## 3. Results and Discussions

### 3.1. Vibrating System Magnetometer (VSM) Measurements

**Figure 1:**
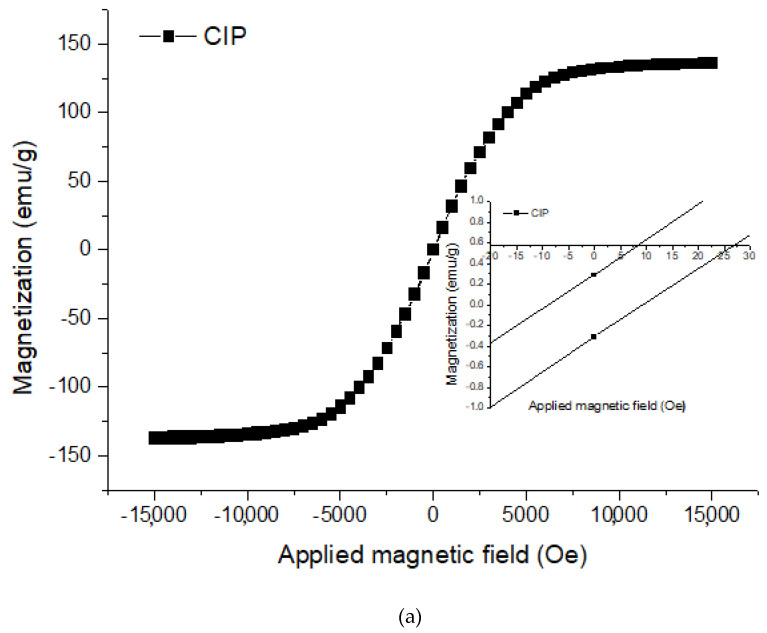

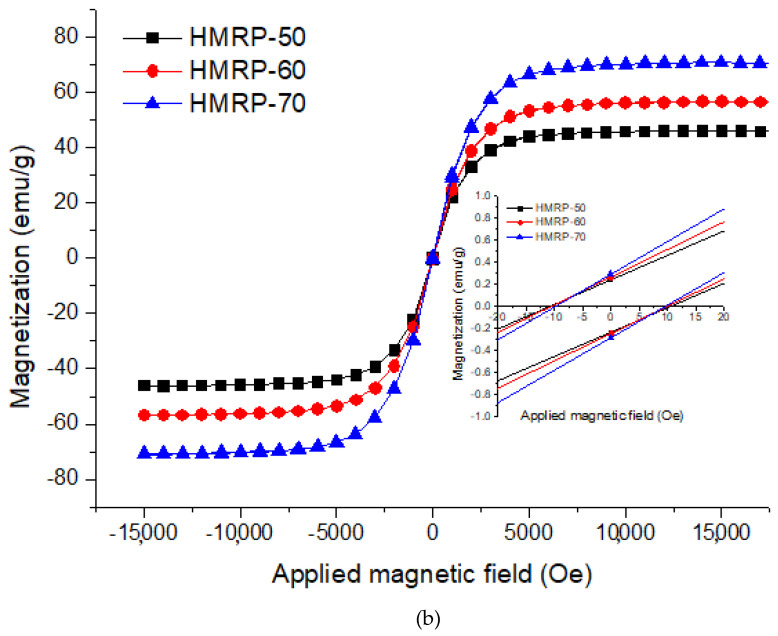
Magnetization curves of (**a**) bare carbonyl iron particles (CIPs) and, (**b**) polyvinyl alcohol hydrogel magnetorheological plastomer (PVA HMRP) samples with different CIPs content.

The magnetization measurements of CIPs and PVA HMRP samples with varying concentrations of CIPs are shown in Figure 1a,b, respectively. Hence, all magnetic parameters such as coercivity (Hc), retentivity (Mr), and magnetic saturation (Ms) were analysed from the hysteresis curve as listed in Table 1.

Based on the magnetic measurement in Figure 1, the PVA HMRP samples show a narrow hysteresis loop and low *H_c_*, characterised by a good soft magnetic property. Moreover, as the magnetic field increased, the magnetization curve increased accordingly and approaches to the saturated plateau region as represented by *M_s_*. From Table 1, the *M_s_* of HMRP-50, HMRP-60, and HMRP-70 are 46.16, 54.74 and 70.92 emu/g, respectively. While for the CIPs, the Ms is 137.06 emu/g.

Furthermore, *M_s_* of the samples increased with increasing of CIPs content. The highest *M_s_* sample (HMRP-70) demonstrated the highest magnetic response to the applied magnetic field. Furthermore, as shown in Table 1, the value of *M_r_* for all samples is small enough to be neglected. The low *M_r_* value indicates that the magnetic moment of the material is lost after removing the magnetic field. The results implied a low remnant magnetisation that was one of the criteria required in the preparation of smart magnetic materials so that the magnetic particles did not remain sticky after the magnetic field was removed. Meanwhile, *H_c_* is defined as the amount of magnetic field required to bring magnetization back to zero. The small value of *H_c_* is one characteristics of good soft magnetic properties of CIPs, which is desirable in fabrication of smart magnetic materials as the material is easily demagnetized after the magnetic field is removed. It is also well known that the *H_c_* value is strongly dependent on the particle orientation in the matrix which mean the particles rotate in the matrix materials [27]. As shown in Table 1, the value of *H_c_* value decreases with increasing concentration of CIPs. When the concentrations of CIP decrease, the distance between the particles is far enough to make it harder for the particles to magnetize compared to the higher concentrations of the sample. Therefore, from Table 1, HMRP-70 shows the lowest *H_c_* value, indicating that the CIPs are easy to orient and magnetize inside the matrix. For lower concentrations of CIPs, the rotation may be hindered by the increased formation of complexes between borate ions and hydroxyl functional groups of PVA solution.

### 3.2. Rheological Properties: Rotational Mode

**Figure 2:**
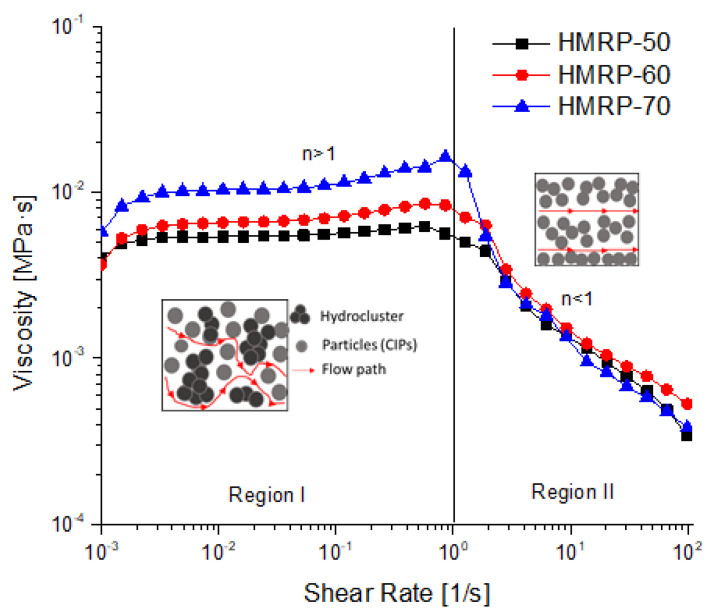
The viscosity versus shear rate for PVA HMRP samples with different CIP contents.

The effects of CIPs content and shear rate on the viscosity of the samples; HMRP-50, HMRP-60 and HMRP-70, are shown in Figure 2 in double logarithmic coordinates. All data were fitted with Equation (1) derived from the Herschel–Bulkley rheological model [28].

(1) $$\eta =\tau_{0}/\dot{\gamma }+k{\dot{\gamma }}^{n-1}$$

where η is apparent viscosity,$\dot{\gamma }$ is the shear rate, k is consistency index, $\tau_{0}$ is derived from dynamic yield stress, and n is the power-law exponent or flow behaviour index. Herschel–Bulkley is known for its capability to determine whether the fluid is shear thickening or thinning by observing the value of n. The values of n<1, n>1, and n=1 represent shear thinning, shear thickening, and Bingham plastic behaviour, respectively.

Figure 2 displays the variation of viscosity with the shear rate for PVA HMRP samples with different CIP contents. In all cases, the viscosity of the PVA HMRP increased with an increase of CIP content. For example, the viscosity for HMRP-50, HMRP-60 and HMRP-70 at a shear rate of 0.01 s^−^ were 0.005, 0.006, and 0.010 MPa.s, respectively. The PVA HMRP samples demonstrated a change in viscosity as two distinct stages (Region I, and Region II) as shown in Figure 2. The flow behaviour of the PVA HMRP could be summarized as follows: Region I (*γ* < 1; very low shear rate), a shear-thickening behaviour exists (*n* > 1) and Region II (*γ >* 1), a shear-thinning behaviour exists (*n* < 1). The fitted flow behaviour index for each sample is listed in Table 2 to understand more about the influence of CIPs content on the flow behaviour of the PVA HMRP.

As depicted in Table 2, the fitting *n*-parameters for HMRP-50, HMRP-60, and HMRP-70 in Region I, at a very low shear rate of 0.001 to 0.1 s^−1^, were 1.06, 1.11 and 1.13, respectively. The results indicated an increase in *n* values with increase in CIPs content. The HMRP-70 sample showed the highest shear-thickening exponent, *n* = 1.13 among the samples. At this region, all viscosities were slightly increased (shear-thickening) due to re-formation of the hydrogen bonding and the chain entanglement between the hydroxyl groups of PVA molecules [29]. Moreover, the increased in viscosity with the increasing shear rate was a consequence of the jamming cluster’s formation or particle aggregation bound together by hydrodynamic forces as shown in the schematic illustration (Figure 2). As shown in the schematic illustration, in Region I the CIPs are forced into proximity and formed hydro clusters resulting in shear thickening behaviour. Moreover, with the increased CIPs, the shear-thickening parameter (*n*) showed an increase from 1.06 to 1.13 as shown in Table 2. The increase in CIP content would lead to a more remarkable shear thickening behaviour as the distance between the particles decreases. As a result, the hydrodynamic forces between the particles would increase and cause more CIPs to aggregate. Therefore, HMRP-70 had the highest value of the flow behaviour index (*n*) of 1.13.

Nonetheless, at a much higher shear rate, abruptly decreased viscosity (shear thinning) was observed in Region II. The shear-thinning parameter (*n*) indicated an increase from 0.42 to 0.70 because the polymer chains of the PVA were completely disintegrating due to a reduction of hydrogen bonding and chain entanglement. After all, they have not been able to keep up with the higher force of shear rate. At this point, the aggregation of CIPs was destroyed, and the flow restriction was decreased, resulting in shear thinning behaviour. However, Wu et al. [22] found that the physically crosslinked PVA hydrogel displayed shear-thinning behaviour along with an increase of shear rate. This differs from the findings presented here, which may be due to the presence of a borax solution as a crosslinking agent that influences the shear-thickening behaviour that existed in this current study. Park et al. [30], also confirmed that the PVA solution with the addition of borax displayed strain-hardening behaviour.

### 3.3. Rheological Properties: Oscillatory Mode

**Figure 3:**
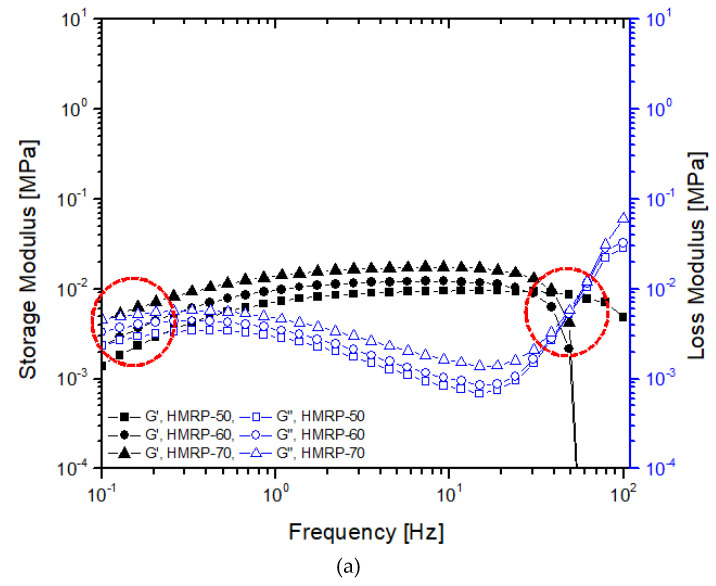

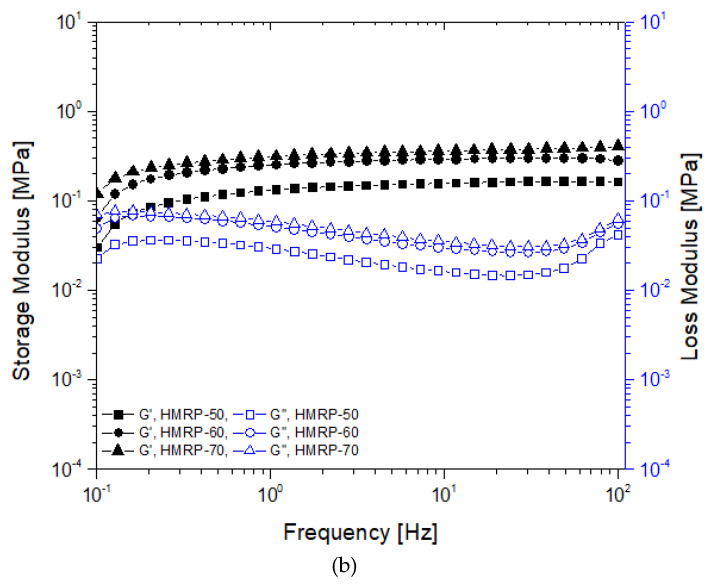

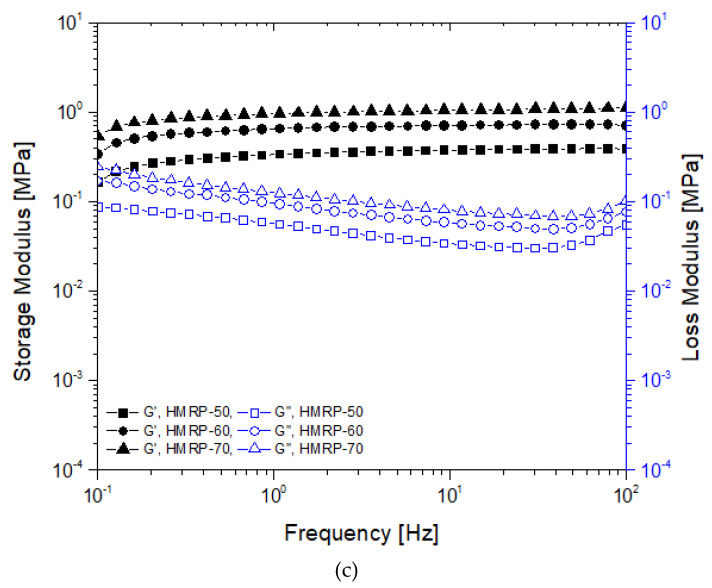

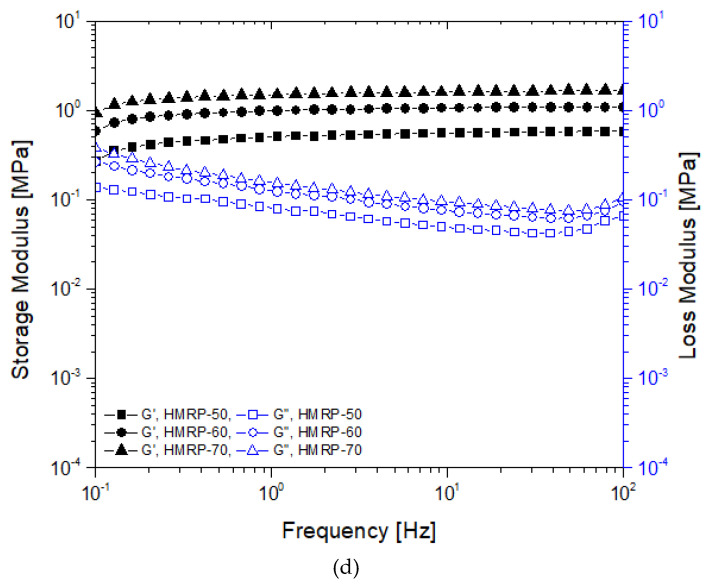
Storage (solid) and loss (open) moduli for PVA HMRP samples as a function of the frequency with different CIP contents at the magnetic field of (**a**) 0, (**b**) 180, (**c**) 370, and (**d**) 540 mT.

Oscillatory shear mode testing was conducted by performing the frequency sweep test with increasing frequency in the range of 0.1 to 100 Hz. The frequency sweep was performed within the linear viscoelastic range at a fixed shear strain amplitude (γ_0_ = 0.03%), The values of storage modulus, *G′* and loss modulus, *G”* were obtained from this measurement. The frequency sweep test was conducted using magnetic fields of different intensities (0, 180, 370, and 540 mT) and is demonstrated in Figure 3a–d with different CIPs contents.

As seen in Figure 3a, in an off-state condition (B = 0 mT), a similar trend is observed for all samples, where *G′* increases abruptly with increasing frequency until it eventually falls at maximum frequency, *f_max_*. For example, the *f_max_* for HMRP-50 was at 50 Hz, while for HMRP-60 and HMRP-70, the *f_max_* shifted to lower than 50 Hz at about *f_max_* = 30 Hz for both. Under the off-state condition, the G′ value depended mainly on the behaviour of the polymer networks. The polymer networks of PVA HMRP were linked by the hydrogen bonding of OH groups from PVA-borate ions, which was a very weak and reversible chemical crosslinking. As a result, with the increasing CIPs content, the polymer networks of the PVA HMRP would be easier to detangle due to aggregations of CIPs. Therefore, HMRP-60 and HMRP-70 samples with a higher CIPs content should have lower *f_max_* value. Moreover, as seen in Figure 3a, above *f_max_*, the G′ would abruptly drop when the polymer networks started to break down because there was not enough time to rearrange and cope with a higher shear frequency.

On the other hand, a unique phenomenon was observed in samples tested at 0 mT (off-state condition) as shown in Figure 3a. Two cross-over points for *G′* and *G”* occurred at low frequency and high frequency, as shown in. Furthermore, in all cases, at low-frequency range (< 0.3 Hz) and at high frequency (>40 Hz) when B = 0 mT, the *G”* for all samples was greater than *G′.* The result indicated the occurrence of liquid-like viscoelastic behaviour of the materials. However, at the middle range (0.3 to 40 Hz), the G′ values were larger than the G” values, which indicated the solid-like viscoelastic behaviour. This phenomenon may be attributed to the reaction of borate ions with the PVA molecular chains as borate ions were believed to control the motion of polymer molecular chains [26,31]. Basically, at off-state condition, the viscoelastic behaviour of the samples depended mainly on the polymer matrix. However, in this study when the CIPs were introduced into the matrix, the effect of the CIPs should be considered. For example, as the CIPs fraction increased, the cross-over point at lower frequency began to shift towards lower frequency (HMRP-60) and diminished (HMRP-70). At this point, it was noted that the CIPs content affected the viscoelastic materials of HMRP, because the CIPs were denser, where the material exhibited more solid-like behaviour rather than liquid-like behaviour. Similar to the function of borax crosslinking, CIPs enhanced the viscosity of the material by forming network structures. The structures of the network formed between the CIPs may induce chain entanglement and restricted the movement of PVA chain motions resulting in solid-like (hardening) behaviour [32].

Meanwhile, different behaviours were observed for all samples tested in the presence of the magnetic field in Figure 3b–d compared to non-magnetic field samples studied. At the on-state condition (B ≠ 0 mT) in all cases, G′ increased slightly with increasing frequency up to 100 Hz. For example, the G′ of CIP-70 (Figure 3d) increased from 1.366 to 1.693 MPa with the change in shear frequency starting from 0.1 to 100 Hz at 540 mT. The increment of G′ along with the increased in frequency indicated that the samples exhibited typical shear hardening shear frequency [33]. Moreover, it was noted that at the initial storage modulus, G_0_ increased with the increased of the external magnetic field, for all samples. When the magnetic field increased, the G_0_ was also increased as the result of particle strengthening effect, in which the CIPs began to form chain-like structures and caused samples to harden. In addition, the CIPs content influenced the strengthening behaviour of the samples. For instance, as shown in Figure 3d, with the increment of the CIPs content from 50 to 70 wt.%, the initial storage modulus, G′ increases from 0.27 to 0.93 MPa, which must be associated to the strengthening effect of CIPs. With the higher CIPs content in the HMRP matrix, the chain structures became denser and complexed, consequently blocking the movement of polymer chains during shear deformation [7]. Furthermore, in the presence of a magnetic field (Figure 3b–d), the HMRP samples present a solid-like viscoelastic behaviour, where G′ > G” values demonstrated a main elastic property. This may be attributed to the strengthening of the particle chains by the application of a magnetic field, which allowed the materials to become stiffer and more elastic (solid-like behaviour). Also, the borate ion can also be considered to be elastically active cross-linkers that caused to the increased behaviour [34].

**Figure 4:**
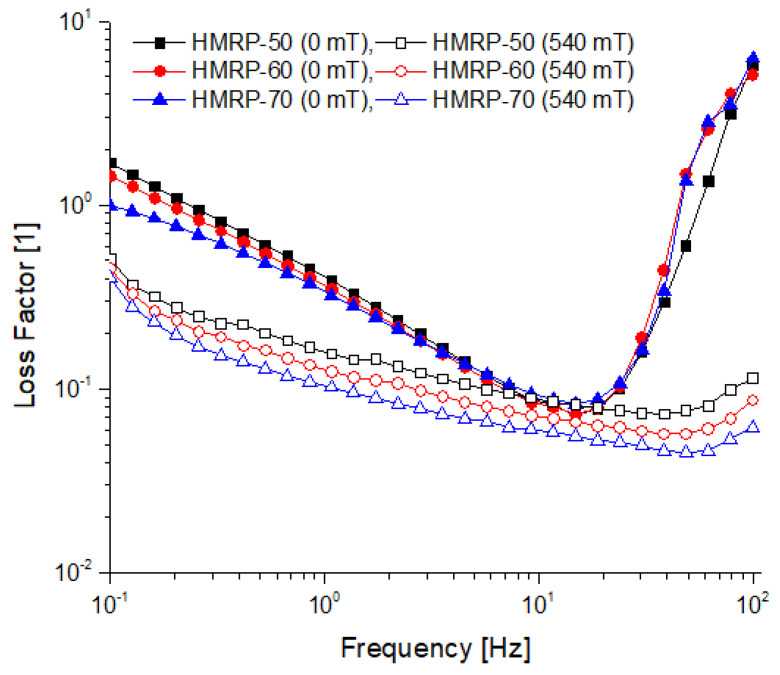
The loss factor of all samples at off-state and on-state conditions.

The loss factor curves that represents the mechanical damping of the viscoelastic material is shown in Figure 4. The loss factor represents the ratio of dissipated energy stored to retained energy (tan δ = G”/G′) during the deformation of materials.

According to An et al. [35], the loss factor reflected the difference in strength between the loss and storage moduli. The low loss factor value suggested that the elastic behaviour (solid-like) was more prevalent than the viscous (liquid-like) nature of the material. From the plot in Figure 4, it is apparent that the tan δ at on-state (B = 540 mT) is higher than of the tan δ at off-state condition (B = 0 mT). Moreover, when B = 0 mT, the initial values (*f* = 0.1 Hz) of tan δ for all samples were greater than 1 and began to decrease to below 1 for the entire frequency range up to *f*~20 Hz. After *f* > 20 Hz, the tan δ suddenly increased again. The factor that predominantly influenced this type of behaviour was due to the entanglement and disentanglement of polymer chains during dynamic stretching which, in turn, was associated with the energy release mechanism in the system. Meanwhile, at higher frequencies (*f* > 20 Hz), the sudden increased in tan δ or dissipation energy could be due to the permanent weakening and breakdown of the polymer chains. This was because the PVA polymer chains begin to rupture at a higher frequency due to a higher force as the chains were interlinked with weak crosslinkers (borax).

Nevertheless, in the presence of external stimuli such as magnetic field (B = 540 mT), the loss factor value was lower than 1 (tan δ < 1) from the start (*f* = 0.1 Hz), suggesting the elastic nature (solid-like behaviour) of the samples under the influence of the external magnetic field. The elastic nature of the sample may be attributed to the strengthening of the CIPs that tended to travel and form chain-like structures along the direction of the magnetic field. Consequently, the distance between the particles was reduced, which led to decrease in tan δ. Moreover, the decrease in tan δ also symbolizes the less dissipation of friction energy due to the stronger magnetic force interaction between the particles. The loss factor or damping of the viscoelastic material was mainly due to the movement of the polymer networks in the matrix and the particle-to-particle friction. Furthermore, the increased in the CIPs content was also led to a decreased in the loss factor in both off-state and on-state conditions. When the HMRP samples became denser with a high CIPs content, the surface contact between the particles was improved that lower the internal friction resulting in the decreased of energy dissipation.

### 3.4. Magnetorheological Effect of PVA HMRP

**Figure 5:**
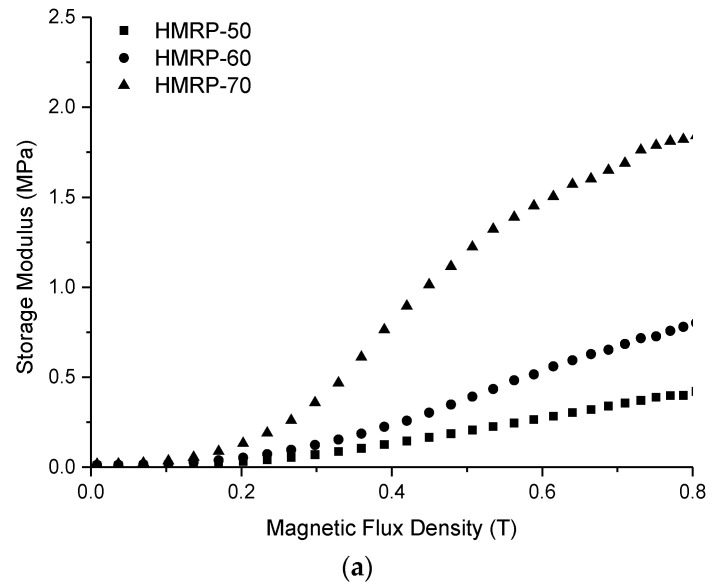

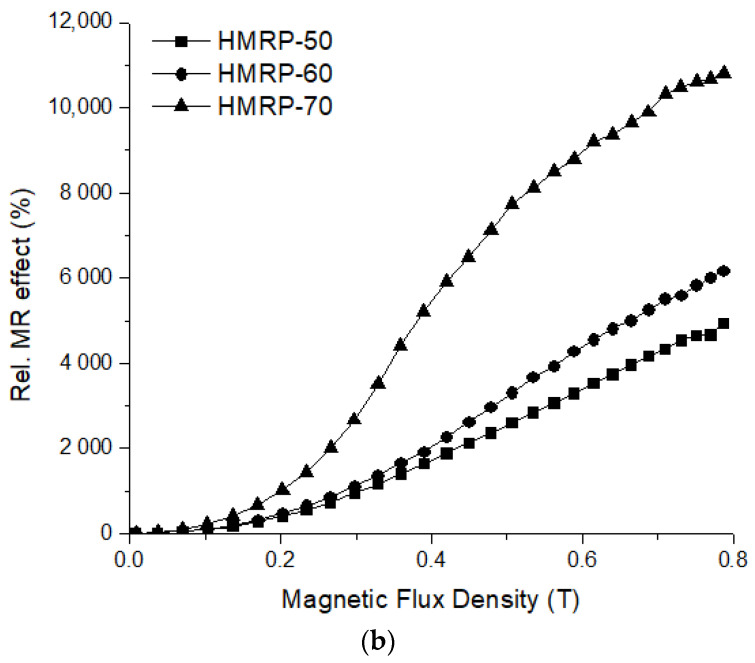
(**a**) Storage moduli and (**b**) relative magnetorheological (MR) effect of PVA HMRP samples with different CIP contents under different magnetic flux densities.

The curve of shear storage modulus (G′) with a magnetic field strength of PVA HMRP samples with different CIPs contents is displayed in Figure 5. The relative and absolute MR effects of each sample can be calculated using the respective equation below:

(2) $$Absolute\;MR\;effect,\;∆G^{\prime }={G^{\prime }}_{max}-{G^{\prime }}_{0}$$

(3) $$Relative\;MR\;effect=\frac{{G^{\prime }}_{max}-{G^{\prime }}_{0}}{{G^{\prime }}_{0}}\times 100\%.$$

where G′_0_ is the zero-field modulus, and G′_max_ is the maximum modulus achieved in the presence of a magnetic field.

As observed in Figure 5, the G′ of all samples show an increasing pattern with the increase in magnetic flux density from 0 to 800 mT. The curve reveals that the CIPs content has a great influenced on G′, where the sample with a higher loading of CIPs (HMRP-70) exhibited notable changes at the highest G′. For instance, at B = 800 mT, the maximum G′ of samples HMRP-50, HMRP-60, and HMRP-70 were 0.4130, 0.7892 and 1.8243 MPa, respectively. Table 3 lists all important parameters such as *G′_0_, G′_max_*, ∆*G′*, absolute and relative MR effect of each sample.

Table 3 shows that all values of *G′_0_*, *G_max_*, ∆*G′* and relative MR effect increase with the increase in CIPs content. The increment of ∆*G′* was caused by further interactions between magnetic particles (CIPs) that have magnetised due to the application of an external magnetic field. Meanwhile, the increase in *G′_0_* was due to an increase in CIPs content, which caused the sample to become harder and denser. According to Liu et al. [36], instead of a cross-linking bond, the CIPs concentration has also influenced the MR behaviour of the materials, and dominantly affected the MR effect. By increasing the concentration of CIPs in the matrix, the space for the movement of all molecular chains would be restricted due to a decrease in the space available for CIPs movement.

Additionally, from the increase in *G′_max_* and ∆*G′*, the results were associated with the highest *M_s_* of the samples as shown in Figure 1. PVA HMRP samples that contained a higher CIPs exhibited a higher *M_s_* because when the magnetic field was applied, the magnetic moments of the CIPs in the matrix tended to orient and form chain-like structures parallel to magnetic field. Consequently, samples with higher CIP contents had higher magnetization and stronger chain-oriented structures, resulting in more remarkable changes in ∆G′, which led to a higher relative MR effect. In the previous study by Wu et al. [22], the relative MR effect of physically crosslinked PVA hydrogel with 70 wt.% of CIPs was reported to be 230%. Compared to this, the chemically crosslinked PVA HMRP prepared in this study has a higher relative MR effect, which was almost ~5 times higher than the previous study. The above results have proved that the chemically crosslinked PVA HMRP has good criteria to be used as one of the smart responsive materials.

## 4. Conclusions

In this study, three samples of chemically crosslinked PVA HMRP containing different CIP contents, HMRP-50, HMRP-60, and HMRP-70 were prepared and the rheological properties of PVA HMRP were successfully investigated. Both rotational and oscillatory shear tests were conducted using a rheometer to study the field-dependent rheological behaviours of the PVA HMRP samples. The CIPs content influences the rheological properties of PVA HMRP either in rotational or oscillatory tests. The viscosity of the samples increased with an increased in the CIPs content. The highest viscosity achieved by the PVA HMRP with a CIPs content of 70 wt.% was 0.062 MPa.s. Moreover, PVA HMRP exhibited different flow behaviours (shear-thinning/shear-thickening) depending on the shear rate. Based on the frequency dependence test, it has been proven that the PVA HMRP displayed shear-thickening behaviours as the storage modulus continues to increase with increasing frequency. The storage modulus and relative MR effect could be adjusted by controlling the strength of the external magnetic field and modifying the CIPs content. With CIP content of 70 wt.%., the maximum storage modulus and the relative MR effect achieved by the PVA HMRP samples were ~1.84 MPa and 10,794%, respectively. The highest relative MR effect was due to movable CIPs that formed strong chain-like structures when subjected to a high magnetic field.

## Figures and Tables

**Table 1:** Magnetic parameters from the magnetization curves.

Sample	Filler Content [wt.%]	*H_c_* [Oe]	*M_s_* [emu/g]	*M_r_* [emu/g]
**HMRP-50**	50	10.84	46.16	241.57 × 10^−3^
**HMRP-60**	60	10.22	56.74	255.50 × 10^−3^
**HMRP-70**	70	9.79	70.92	290.43 × 10^−3^
**CIPs**	null	9.163	137.06	302.42 × 10^−3^

**Table 2:** The shear-thinning parameter, *n*, fitted based on the Herschel–Bulkley model.

CIPs Content (wt%)	Flow Behaviour Index, *n*	
	Region I	Region II
**50**	1.06	0.42
**60**	1.11	0.65
**70**	1.13	0.70

**Table 3:** The initial storage (G′_0_), maximum storage (G_max_), magneto-induced storage modulus (∆G′) and relative MR effect values of PVA HMRP samples.

PVA HMRP	G′_0_ [MPa]	G′_max_ [MPa]	Magneto-Induced, ∆G′	Relative MR Effect
**HMRP-50**	0.0084	0.4214	0.4130	4916%
**HMRP-60**	0.0128	0.8020	0.7892	6165%
**HMRP-70**	0.0169	1.8412	1.8243	10,794%
